# TAS-102 (trifluridine/tipiracil) plus bevacizumab versus TAS-102 alone as salvage treatment options for metastatic colorectal cancer in routine clinical practice

**DOI:** 10.3389/fonc.2024.1450732

**Published:** 2024-09-30

**Authors:** Ji Eun Shin, Sung Hee Lim, Jeeyun Lee, Ho Yeong Lim, Young Suk Park, Seung Tae Kim

**Affiliations:** ^1^ Division of Medical Oncology, Department of Internal Medicine, St. Vincent’s Hospital, College of Medicine, Catholic University of Korea, Seoul, Republic of Korea; ^2^ Division of Hematology-Oncology, Department of Medicine, Samsung Medical Center, Sungkyunkwan University School of Medicine, Seoul, Republic of Korea

**Keywords:** metastatic colorectal cancer, salvage treatment, TAS-102, bevacizumab, TAS-102 plus bevacizumab

## Abstract

**Introduction:**

Both regimens of TAS-102 (trifluridine/tipiracil) with and without bevacizumab are considered standard options for salvage treatment in patients with refractory metastatic colorectal cancer.

**Materials and methods:**

This analysis included patients with metastatic colorectal cancer who received either TAS-102 plus bevacizumab or TAS-102 alone between July 2022 and November 2023 at Samsung Medical Center. We evaluated the objective response rate (ORR), progression-free survival (PFS), overall survival (OS), and safety profile of both regimens.

**Results:**

In total, 139 patients were included in this analysis. Median age was 60.8 years, and median number of previous lines of therapy was four (range: 2.45–6.55). More than half of the subjects (56.8%) had RAS mutations and 92.9% received previous anti-VEGF therapy. 83 (59.7%) patients received the combination of TAS-102 and bevacizumab and 56 (40.3%) received TAS-102 alone. The number of patients with prior regorafenib treatment was 14 in the TAS-102 with bevacizumab group and 5 in the TAS-102 alone group. The disease control rate was 51.8% in the combination group and 32.1% in the TAS-102 alone group. The median PFS was 3.3 months (95% CI, 2.7–6.6) in the combination group and 2.5 months (95% CI, 2.0–3.8) in the TAS-102 alone group (HR, 0.56; 95% CI, 0.38–0.82; p=0.003). The median OS in these two groups was 10.8 months (95% CI, 8.4–NA) and 6.0 months (95% CI, 4.8–9.8), respectively (HR, 0.62; 95% CI, 0.40–0.97, p=0.033). In the exploratory analysis of TAS-102 + Bev group, patients with the KRAS G12 mutation had inferior OS compared to those without the mutation (HR, 2.01, 95% CI, 1.04–3.90, *p*=0.035). Commonly observed adverse events were hematologic-related, including neutropenia, anemia, and thrombocytopenia, as well as nausea. While any grade neutropenia was observed at similar frequencies in the two groups (57.8% and 57.1%), grade 3 or higher neutropenia was more frequent in the combination group than the TAS-102 alone group (31.3% vs. 17.9%). Among patients who received subsequent anticancer therapy after treatment failure, 74.1% received regorafenib.

**Conclusions:**

The combination of TAS-102 and bevacizumab resulted in a better survival outcome than TAS-102 monotherapy, consistent with previous studies. This analysis supports the use of the combination of TAS-102 and bevacizumab as the best therapeutic option for patients with refractory metastatic colorectal cancer in clinical practice.

## 1 Introduction

Colorectal cancer (CRC) is the fourth most commonly diagnosed cancer and the second leading cause of cancer-related deaths (1). Approximately 15–30% of patients present with metastases at diagnosis, and 20–50% of initially localized cases eventually develop metastases (2). Management of metastatic CRC involves various active drugs used in combination or as single agents. Standard first-line treatment includes a backbone of 5-fluorouracil (5-FU) with the addition of oxaliplatin or irinotecan, often combined with EGFR-targeted antibodies like cetuximab or panitumumab or antiangiogenic therapies like bevacizumab, ramucirumab, or aflibercept. Second-line treatment is determined based on the first-line treatment received (3). Despite failure to respond to these treatments, many patients remain medically fit; however, the survival efficacy of subsequent treatments after second-line treatment for CRC is relatively limited, indicating an unmet need for new treatment options (4).

TAS-102 (trifluridine/tipiracil) is an oral combination drug consisting of a cytotoxic thymidine analog, trifluridine, and a thymidine phosphorylase inhibitor, tipiracil hydrochloride, which prevents the degradation of trifluridine (5). RECOURSE, a phase 3 trial, demonstrated that TAS-102 significantly prolonged overall survival compared with placebo in patients with metastatic CRC who had undergone extensive prior treatments, even in patients with disease refractory to fluoropyrimidines, across patient subgroups of age, geographical origin, or RAS mutation status (7.1 vs. 5.3 months; HR, 0.68; 95% CI, 0.58–0.81; p <.001) (6).

Bevacizumab is a humanized monoclonal antibody that blocks the activity of VEGF, important in tumor angiogenesis (7). Bevacizumab has been proven to improve outcomes when combined with cytotoxic chemotherapy as a first-line treatment for metastatic CRC (8–10). Moreover, continuation of bevacizumab following progression has also been shown to provide a survival benefit (11, 12).

The combination of TAS-102 with bevacizumab has been clinically demonstrated to provide a survival benefit compared to either drug alone after failure of standard therapy in metastatic CRC, and this combination is used in clinical practice. C-TAST FORCE, a phase 2 trial, evaluated the survival efficacy of TAS-102 plus bevacizumab and reported a 16-week progression-free survival rate of 42.9% (80% CI, 27.8–59.0%) in CRC patients who were refractory or intolerant to fluoropyrimidine, irinotecan, oxaliplatin, anti-VEGF therapy, and anti-EGFR therapy (for tumors with wild-type KRAS) and who had no previous treatment with regorafenib (13). Consistent with these results, SUNLIGHT, a phase 3 trial, showed a significant survival benefit in patients who had received no more than two previous chemotherapy regimens for the treatment of advanced CRC. The median overall survival was 10.8 months (HR, 0.61; 95% CI, 0.49–0.77; p<0.001), and the median progression-free survival was 5.6 months (HR, 0.44; 95% CI, 0.36– 0.54; p <0.001) (14).

Regimens comprising TAS-102 with and without bevacizumab are considered standard salvage treatment options in patients with refractory metastatic CRC. In this study, we evaluated the efficacy and safety of TAS-102 with and without bevacizumab in patients with refractory metastatic CRC in regular clinical practice.

## 2 Materials and methods

### 2.1 Study design and participants

This analysis included patients with metastatic colorectal cancer who received either TAS-102 plus bevacizumab or TAS-102 between July 2022 to November 2023 at Samsung Medical Center. We reviewed electronic clinical records to gather information including age, sex, Eastern Cooperative Oncology Group (ECOG) performance status, histopathology, MSI status, RAS mutation status, BRAF mutation status, tumor mutation burden (TMB), carcinoembryonic antigen (CEA) level, primary tumor location, sites of metastatic disease, and previous treatments. MSI status, RAS mutation status, BRAF mutation status, and TMB were identified using next-generation sequencing (NGS) with the TruSight Oncology 500 assay (Illumina, San Diego, CA, USA) and Oncomine Focus assay (ThermoFisher, Waltham, MA, USA). Patients were treated with TAS-102 35 mg/m² orally twice a day on day 1-5 and 8-12 in a 28-day cycle with or without bevacizumab (5 mg/kg intravenously) every two weeks.

### 2.2 Outcomes

Clinical outcomes evaluated were objective response rate (ORR), disease control rate (DCR), progression-free survival (PFS), and overall survival (OS). Tumor response was evaluated as complete response (CR), partial response (PR), stable disease (SD), or progressive disease (PD), according to the Response Evaluation Criteria in Solid Tumor, version 1.1. ORR was defined as the percentage (%) of patients with confirmed CR or PR. DCR was defined as the percentage (%) of patients with confirmed CR, PR, or SD. PFS was measured from the start of the treatment to the date of disease progression or death from any cause using RECIST 1.1. OS was calculated from the start of the treatment to the date of death from any cause. Safety objectives were evaluated according to Common Terminology Criteria for Adverse Events (CTCAE) version 4.03.

### 2.3 Statistical analysis

Categorical and continuous variables were summarized using descriptive statistics. Survival analyses were performed using Kaplan-Meier curves and compared using log-rank tests. Cox proportional hazards regression models were used to obtain estimates of hazard ratios (HRs) based on multivariate analysis. All P values were two-sided and confidence intervals (CI) were at the 95% level, with statistical significance defined as P ≤ 0.05. All statistical analyses were performed using IBM SPSS Statistics 27 and R version 4.3.0. The data cutoff date was February 28, 2024.

## 3 Results

### 3.1 Clinical characteristics

83 patients received TAS-102 plus bevacizumab (TAS-102 + Bev) and 56 received TAS-102 alone (TAS-102).

Baseline characteristics of analyzed patients are presented in **Table 1**. The median age was 60.8 years, and 56.8% of patients were male. Almost all patients (95.0%) were MSS, while only two (1.4%) were MSI-H, both of whom received TAS-102 + Bev. Among patients, 56.8% had RAS mutations, and six (4.3%) had BRAF mutations. The median TMB was 6.9, with 24 patients (17.3%) having a TMB-H status (>10 mutations). Primary tumor location was on the left side in 80.6% of patients, of whom 55 (39.6%) had tumors in the rectum. Metastases to the liver, lung, bone, peritoneum, and other sites were observed in 67.6%, 73.4%, 5.8%, 29.5%, and 42.4% of patients, respectively. The median number of previous lines of therapy was four (range: 2.45–6.55). All patients had previously received 5-FU, with 96.4% and 98.6% having received oxaliplatin and irinotecan, respectively. One hundred thirty-four patients had received anti-VEGF therapy (106 patients received bevacizumab only, 27 received both bevacizumab and aflibercept, and one received aflibercept only). Anti-EGFR therapy had been administered to 36 patients (25.9%). Fourteen patients in the TAS-102 + Bev group had prior regorafenib treatment compared to five in the TAS-102 group.

**Table 1:** Baseline Characteristics.

Patient Characteristics	Total (n=139)	TAS-102 + Bev (n=83)	TAS-102 (n=56)	*p*-value
Age (years), median (range)	60.8 ± 11.1	58.6 ± 10.6	64.1 ± 11.2	0.004
Age < 65	90 (64.7%)	63 (75.9%)	27 (48.2%)	0.002
Age ≥ 65	49 (35.3%)	20 (24.1%)	29 (51.8%)	
Sex				0.351
Male	79 (56.8%)	44 (53.0%)	35 (62.5%)	
Female	60 (43.2%)	39 (47.0%)	21 (37.5%)	
ECOG performance status				0.012
0	40 (28.8%)	31 (37.3%)	9 (16.1%)	
1	90 (64.7%)	49 (59.0%)	41 (73.2%)	
≥ 2	9 (6.5%)	3 (3.6%)	6 (10.7%)	
Differentiation				0.956
Well differentiated	25 (18.0%)	16 (19.3%)	9 (16.1%)	
Moderately differentiated	102 (73.4%)	60 (72.3%)	42 (75.0%)	
Poorly differentiated	7 (5.0%)	4 (4.8%)	3 (5.4%)	
Signet ring cell	2 (1.4%)	1 (1.2%)	1 (1.8%)	
Unknown	3 (2.2%)	2 (2.4%)	1 (1.8%)	
MSI status				0.257
MSS	132 (95.0%)	78 (94.0%)	54 (96.4%)	
MSI-L	0 (0.0%)	0 (0.0%)	0 (0.0%)	
MSI-H	2 (1.4%)	2 (2.4%)	0 (0.0%)	
MSI-I	2 (1.4%)	2 (2.4%)	0 (0.0%)	
Unknown	3 (2.2%)	1 (1.2%)	2 (3.6%)	
RAS mutation status				0.037
RAS mutant	79 (56.8%)	53 (63.9%)	26 (46.4%)	
KRAS mutant	74 (53.2%)	48 (57.8%)	26 (46.4%)	
NRAS mutant	5 (3.6%)	5 (6.0%)	0 (0.0%)	
RAS wild	51 (36.7%)	24 (28.9%)	27 (48.2%)	
Unknown	9 (6.5%)	6 (7.2%)	3 (5.4%)	
BRAF mutation status				0.361
BRAF mutant	6 (4.3%)	2 (2.47%)	4 (7.1%)	
BRAF wild	125 (89.9%)	76 (91.6%)	49 (87.5%)	
Unknown	8 (5.8%)	5 (6.0%)	3 (5.4%)	
TMB				
TMB	6.9 ± 3.6	7.3 ± 3.6	6.1 ± 3.6	0.484
TMB-L	88 (63.3%)	57 (68.7%)	31 (55.4%)	
TMB-H	24 (17.3%)	18 (21.7%)	6 (10.7%)	
Unknown	27 (19.4%)	8 (9.6%)	19 (33.9%)	
Primary tumor location				
Right side	27 (19.4%)	25 (30.1%)	12 (21.4%)	0.786
Left side	112 (80.6%)	58 (69.9%)	44 (78.6%)	
Rectum	55 (39.6%)	31 (37.3%)	24 (42.9%)	0.635
Site of metastatic disease				
Liver	94 (67.6%)	54 (65.1%)	40 (71.4%)	0.547
Lung	102 (73.4%)	58 (69.9%)	44 (78.6%)	0.346
Bone	8 (5.8%)	5 (6.0%)	3 (5.4%)	1.000
Peritoneum	41 (29.5%)	25 (30.1%)	16 (28.6%)	0.995
Others	59 (42.4%)	36 (43.4%)	23 (41.1%)	0.925
Number of metastatic sites				0.621
1	31 (22.3%)	21 (25.3%)	10 (17.9%)	
2	66 (47.5%)	39 (47.0%)	27 (48.2%)	
3	28 (20.1%)	14 (16.9%)	14 (25.0%)	
≥ 4	14 (10.1%)	10 (10.8%)	5 (8.9%)	
Previous lines of therapy				0.747
1	4 (2.9%)	3 (3.6%)	1 (1.8%)	
2	63 (45.3%)	39 (47.0%)	24 (42.9%)	
3	39 (28.1%)	22 (26.5%)	17 (30.4%)	
4	21 (15.1%)	10 (12.0%)	11 (19.6%)	
5	8 (5.8%)	6 (7.2%)	2 (3.6%)	
6	4 (2.9%)	3 (3.6%)	1 (1.8%)	
Previous history of regorafenib				0.278
Yes	19 (13.7%)	14 (16.9%)	5 (8.9%)	
No	120 (86.3%)	69 (83.1%)	51 (91.1%)	

ECOG, Eastern Cooperative Oncology Group; MSI, microsatellite instability; TMB, tumor mutational burden.

The median age of patients in the TAS-102 + Bev group was 58.6 years, which was younger compared to 64.1 years in the TAS-102 group. The proportion of patients with poor performance status (ECOG 2 or higher) was smaller in the TAS-102 + Bev group (3.6% vs. 10.7%), which contained a larger number of patients with RAS mutations (63.9% vs. 46.4%). Other baseline characteristics were similar between the two groups.

### 3.2 Efficacy of TAS-102 with and without bevacizumab

During the median follow-up duration of 13.8 months (range: 10.0–16.0 months), the median number of treatment cycles was three (range: 1–13) in the TAS-102 + Bev group and two (range: 1–11.5) in the TAS-102 group.

Seven patients achieved PR (8.4%), while 36 achieved SD (43.4%), resulting in an ORR of 8.4% and a DCR of 51.8% in the TAS-102 + Bev group. In the TAS-102 group, three patients (5.4%) had PR, 15 patients (26.5%) had SD, while 28 patients, which is half of the group, achieved PD, resulting in an ORR of 5.4% and a DCR of 32.1% (**Table 2**).

**Table 2:** Objective response rates by treatments.

Treatment response	TAS-102 + Bev (n=83)	TAS-102 (n=56)
No. of cycles, median (range)	3 (1 – 13)	2 (1 - 11.5)
Overall ORR, No. (%)		
Complete response	0 (0.0%)	0 (0.0%)
Partial response	7 (8.4%)	3 (5.4%)
Stable disease	36 (43.4%)	15 (26.8%)
Progression disease	33 (39.8%)	28 (50.0%)
Not evaluated	7 (8.4%)	10 (17.9%)
Objective response rate, %	8.4%	5.4%
Disease control rate, %	51.8%	32.1%

ORR, Objective response rate.

**Figure 1:**
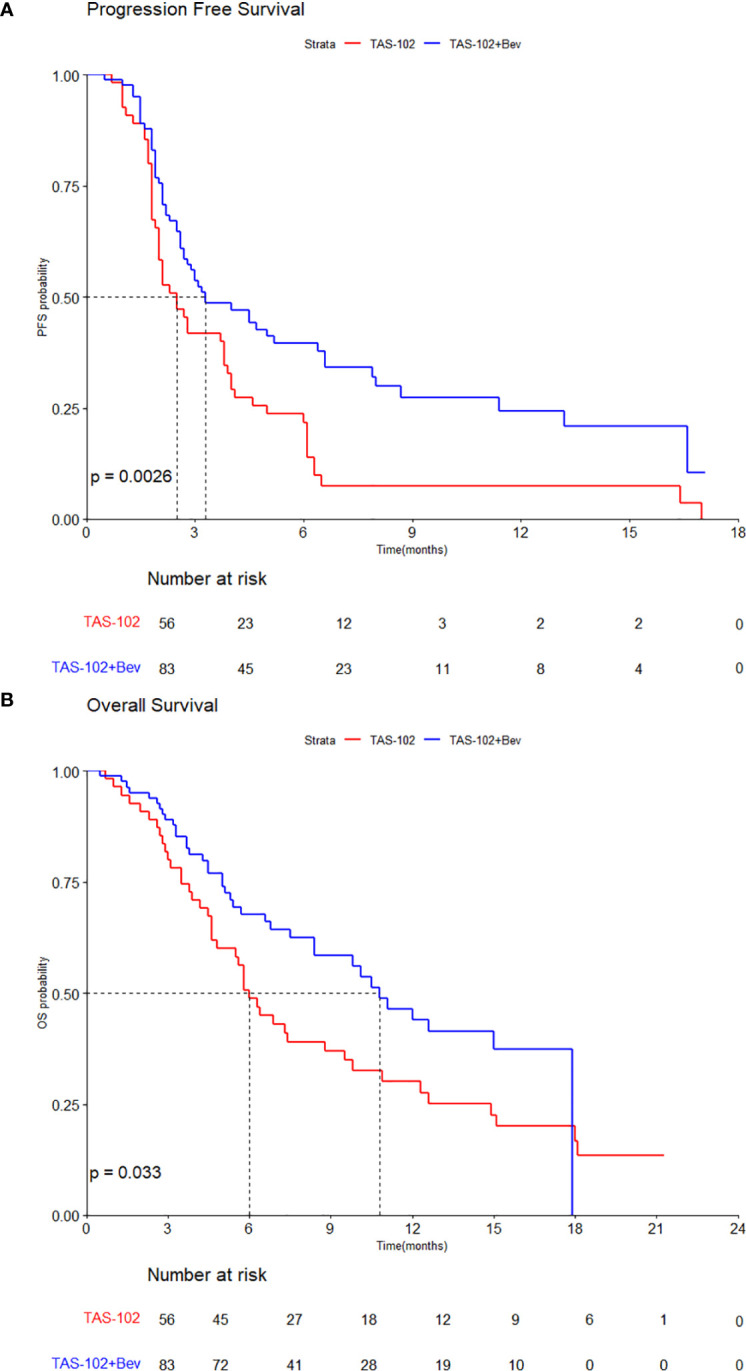
Kaplan–Meier curves by treatments for **(A)** progression-free survival and **(B)** overall survival.

The median PFS of all patients was 3.0 months (95% CI, 2.5–4.0), with values of 3.3 months (95% CI, 2.7–6.6) in the TAS-102 + Bev group and 2.5 months (95% CI, 2.0–3.8) in the TAS-102 group (HR, 0.56; 95% CI, 0.38–0.82; p=0.003) (**Figure 1A**). The median OS was 8.4 months (95% CI, 6.4–11.1) in all patients, with values of 10.8 months (95% CI, 8.4–NA) in the TAS-102 + Bev group and 6.0 months (95% CI, 4.8–9.8) in the TAS-102 group (HR, 0.62; 95% CI, 0.40–0.97, p=0.033) (**Figure 1B**).

**Figure 2:**
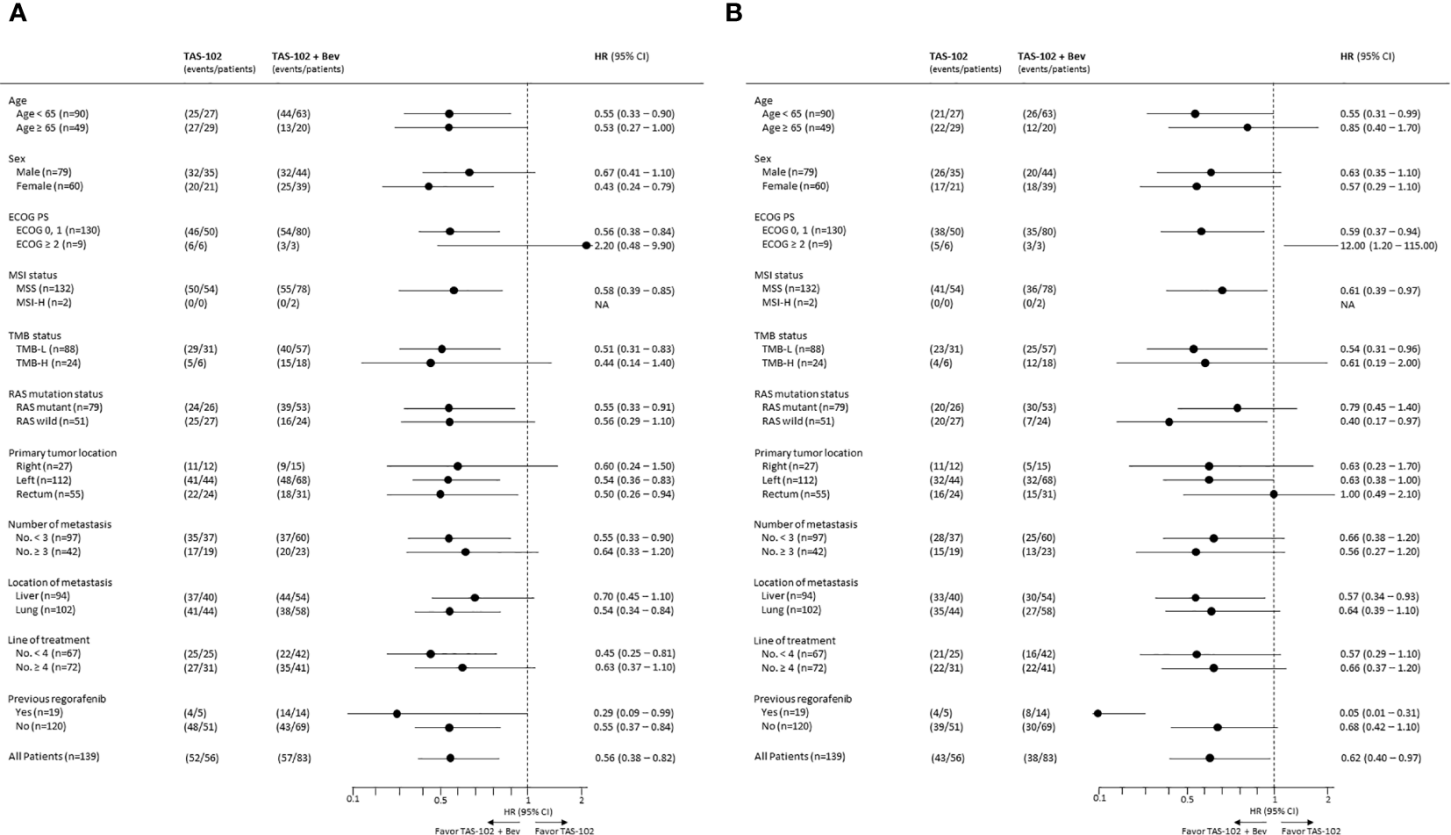
Forest plots of subgroup analyses of **(A)** progression-free survival and **(B)** overall survival.

Subgroup analyses of PFS revealed favorable survival outcomes for most subgroups in the TAS­102 + Bev compared to TAS­102 group, except for patients with an ECOG PS ≥ 2 (**Figure 2A**). Similar to PFS, longer OS was observed in all subgroups who received TAS­102 + Bev treatment, except for the subgroup with ECOG PS ≥ 2 and a primary tumor location in the rectum (**Figure 2B**).

### 3.3 Exploratory analysis of TAS-102 + Bev by KRAS G12 mutation

An exploratory analysis was conducted in the TAS-102 + Bev group to determine whether KRAS mutation status affects the clinical outcome of TAS-102 + Bev. Out of the total 83 patients treated with TAS-102 + Bev, 5 patients whose KRAS mutation status was unknown were excluded. Of these, 35 patients had KRAS G12 mutation (KRAS G12) while 43 had no KRAS G12 mutation (No KRAS G12). Among No KRAS G12, 13 had other KRAS mutation status: 11 had the KRAS G13 mutation, and 2 had other uncommon KRAS mutations.

**Figure 3:**
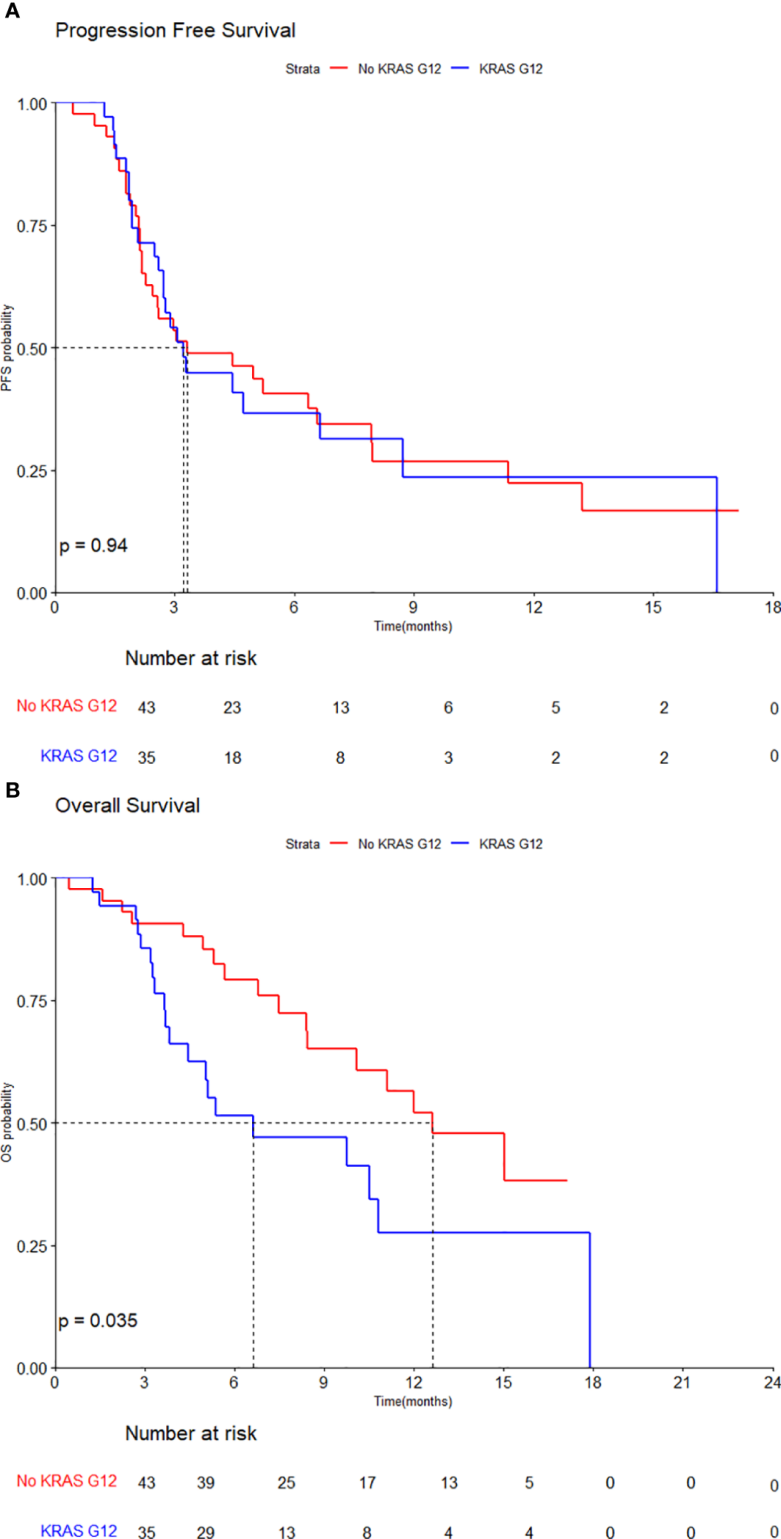
Kaplan–Meier curves by KRAS G12 mutation for **(A)** progression-free survival and **(B)** overall survival.

The median PFS of total was 3.3 months (95% CI, 2.7–6.6), with 3.2 months (95% CI, 2.7–NA) in KRAS G12 group and 3.3 months (95% CI, 2.3–8.0) in the No KRAS G12 group. The PFS of TAS-102 + Bev did not differ significantly by KRAS mutation status (HR, 1.02, 95% CI, 0.60–1.75, *p*=0.94) (**Figure 3A**).

However, the median OS of total population was 10.8 months (95% CI, 10.1–NA), with the median OS of 6.6 months (95% CI, 4.5–NA) in KRAS G12 group and 12.6 months (95% CI, 10.1–NA) in No KRAS G12 group. The OS of TAS-102 + Bev was statistically significantly inferior in patients with the KRAS G12 mutation (HR, 2.01, 95% CI, 1.04–3.90, *p*=0.035) (**Figure 3B**).

### 3.4 Safety profile of TAS-102 with and without bevacizumab

In the two groups, commonly observed adverse events (AEs) were hematologic in nature, including neutropenia, anemia, and thrombocytopenia, as well as nausea (**Table 3**). While any grade neutropenia was observed at similar frequencies in the groups (57.8% and 57.1%), grade 3 or higher neutropenia was more frequently observed in the TAS-102 + Bev group than the TAS-102-alone group (31.3% vs. 17.9%).

**Table 3:** Adverse events by treatments.

Adverse events	TAS-102 + Bev (n=83)		TAS-102 (n=56)	
	Any grade	Grade ≥ 3	Any grade	Grade ≥ 3
Neutropenia	48 (57.8%)	26 (31.3%)	32 (57.1%)	10 (17.9%)
Anemia	63 (75.9%)	9 (10.8%)	53 (94.6%)	10 (17.9%)
Thrombocytopenia	27 (32.5%)	2 (2.4%)	18 (32.1%)	0 (0.0%)
Nausea	16 (19.3%)	0 (0.0%)	19 (33.9%)	1 (1.8%)
Diarrhea	7 (8.4%)	0 (0.0%)	5 (8.9%)	0 (0.0%)
Vomiting	0 (0.0%)	0 (0.0%)	5 (8.9%)	3 (5.4%)
Fatigue	10 (12.0%)	0 (0.0%)	8 (14.3%)	0 (0.0%)
Mucositis	6 (7.2%)	0 (0.0%)	6 (10.7%)	0 (0.0%)
Proteinuria	18 (21.7%)	0 (0.0%)	3 (5.4%)	0 (0.0%)

For non-hematologic AEs, incidence rates were similar between the two groups, except for proteinuria. Grade 3 or higher non-hematologic AEs were rare. Proteinuria was infrequent in the TAS-102 group but occurred in 21.7% of the TAS-102 + Bev group, although all cases were lower than grade 3.

### 3.5 Subsequent treatments after response failure to TAS-102 with and without bevacizumab treatment

Among the 83 patients who received TAS-102 + Bev, 36 underwent subsequent anticancer therapy. Excluding nine patients who received prior regorafenib, 20 of the remaining 27 patients received regorafenib as the subsequent therapy. Of nine patients who had received regorafenib previously, eight were treated with a 5FU-based regimen (five received capecitabine, two received 5-FU/LV, and one received irinotecan/capecitabine), while the remaining patient was re-treated with regorafenib.

## 4 Discussion

In this study, we demonstrated that TAS-102 plus bevacizumab provided a meaningful survival benefit and had a manageable safety profile compared to TAS-102 alone as a salvage regimen in patients with refractory metastatic CRC, consistent with the findings of previous studies. The median PFS in this analysis was relatively shorter than that reported in the SUNLIGHT study. In SUNLIGHT, 92.1% of patients had received only two prior therapies, whereas 51.8% of patients in this analysis had received at least three prior therapies and 23.7% had received four or more prior therapies (14). The differences in patient populations analyzed in the two studies may have contributed to the differences in PFS between these two studies.

TAS-102 with anti-VEGF therapy has been shown to have an anti-tumor effect in preclinical studies. The combination of TAS-102 with bevacizumab increases phosphorylated trifluridine levels within tumors while avoiding an increase in systemic trifluridine exposure (15). This enhances antitumor efficacy and extends survival, highlighting the superiority of TAS-102 plus bevacizumab over TAS-102 alone.

TAS-102 plus bevacizumab had survival benefits in all subgroups except patients with ECOG PS ≥2. Among nine patients with ECOG PS of 2 or higher, three received TAS-102 + Bev treatment, whereas six received TAS-102 only. Most of these patients received the treatment for two or fewer cycles, with a PFS shorter than two months and OS shorter than five months. However, one patient in the TAS-102 group was a notable long survivor with a PFS of 3.8 months and an OS of 18.6 months, which is believed to be the cause of the hazard ratio inversion. In subgroup analysis, patients, irrespective of their RAS mutation status, showed better survival outcomes when treated with TAS-102 + Bev. This finding aligns with previous prospective studies (16). Additionally, patients with prior bevacizumab and regorafenib treatment had favorable survival outcomes after TAS-102 + Bev treatment.

In previous preclinical studies, it has been confirmed that CRC can have different disease entities depending on their codon-specific KRAS mutation (17). Furthermore, recent studies showed that the KRAS G12 mutation can act as a biomarker for reduced OS benefit from TAS-102 treatment in CRC patients (18). In this study, the OS of TAS-102 + Bev was inferior in patients with the KRAS G12 mutation compared to those without the mutation. Similar to its role as a potential biomarker for poor outcomes in TAS-102 treatment of CRC, this study demonstrates that the KRAS G12 mutation can also serve as a biomarker when TAS-102 is combined with bevacizumab. Considering that TAS-102 + Bev is used as a salvage option in metastatic CRC and can be recommended with other options such as regorafenib or capecitabine, selecting patients who would benefit more from TAS-102 + Bev through codon-specific KRAS mutation analysis would be highly advantageous in clinical practice. However, given the small sample size of this cohort, these findings should be interpreted with caution and further prospective study with large cohort will be necessary.

Safety profiles were comparable between the two groups, although a higher incidence of grade 3 or higher neutropenia was observed in the TAS-102 + Bev group than the TAS-102 group. This might be related to increased phosphorylated trifluridine accumulation facilitated by bevacizumab, as suggested by preclinical studies (15). Furthermore, the risk of severe neutropenia in the TAS-102 + Bev group might potentially have been increased by the longer duration of therapy and by concentration of phosphorylated trifluridine in hemopoietic cells.

TAS-102 plus bevacizumab is recommended as one of the standard treatment options in metastatic CRC patients that have progressed following first- and second-line therapies involving 5-FU plus oxaliplatin or irinotecan along with anti-EGFR or VEGFR therapies when excluding biomarker-directed and immunotherapy. Regorafenib is also considered a standard option in this clinical setting. Regorafenib was demonstrated to have efficacy in the CORRECT trial (OS of 6.4 months for regorafenib vs. 5.0 months for placebo; HR, 0.77; 95% CI, 0.64–0.94; p= .005; PFS of 1.9 months vs. 1.7 months; HR, 0.49; 95% CI, 0.42–0.58; p <.000001) and the CONCUR trial (OS of 8.8 months for regorafenib vs. 6.3 months for placebo; HR, 0.55; 95% CI, 0.40–0.77; p <.001) (19, 20). TAS-102 alone and regorafenib have shown similar survival outcomes as third-line or later treatments in heavily pretreated metastatic CRC populations (21–24). However, the common adverse events of the two agents are different, and TAS-102 is better tolerated with fewer severe adverse effects than regorafenib, which can lead to higher adherence and compliance among patients (25). In this study, 74.1% of patients received regorafenib after failure with TAS-102 plus bevacizumab. Further research is needed to evaluate the optimal sequence of salvage therapies with regard to TAS-102 plus bevacizumab and regorafenib (26–28).

This study had several limitations. First, data were obtained from a single center and were retrospective in nature, which could have resulted in bias. In particular, AEs were determined from EMRs, where they may not have been well documented, leading to a possibility of underestimation. Second, there were differences in patient populations between the TAS-102 + Bev and TAS-102 groups. These differences could have affected the survival outcomes. Third, potential interventions such as palliative primary tumor resection, metastasectomy, or radiation therapy during treatment may have interfered with survival outcomes.

The combination of TAS-102 and bevacizumab resulted in better survival outcomes than TAS-102 monotherapy, consistent with previous studies. Our findings support the use of TAS-102 and bevacizumab in combination as the best therapeutic option for patients with refractory metastatic colorectal cancer in clinical practice.

## Data Availability

The original contributions presented in the study are included in the article/supplementary material. Further inquiries can be directed to the corresponding author.
